# Supplementary material for: MetaRibo-Seq measures translation in microbiomes
Source: Nat Commun. 2020 Jun 29;11:3268. doi: 10.1038/s41467-020-17081-z (PMC7324362; doi:10.1038/s41467-020-17081-z)
Supplement: Supplementary file 10 — Supplementary Data 7 [file 41467_2020_17081_MOESM10_ESM.zip › File2/Confidence_VeryHigh_Taxonomy/189845_out.krona.html]

Javascript must be enabled to view this page.

members
magnitude
magnitudeUnassigned
count
unassigned
taxon
rank

189845\_out

8

2
superkingdom
8

8
phylum
1239

186801
7
class

186802
order
7

family
7
186803


SRS015065\_contig\_number\_19164
33042
genus
1
7


SRS015578\_contig\_number\_7024SRS016132\_contig\_number\_5418
1896997
species
2

species
1

SRS011302\_contig\_number\_5518
2293091


SRS011084\_contig\_number\_27987SRS014459\_contig\_number\_25161SRS015431\_contig\_number\_62204
33043
species
3

909932
1
class

909929
1
order

1843491
family
1

1
genus
970

712532

SRS063603\_contig\_number\_contig-100\_3891.195728
1
species
